# A UCMPs@MIL-100 based thermo-sensitive molecularly imprinted fluorescence sensor for effective detection of β-lactoglobulin allergen in milk products

**DOI:** 10.1186/s12951-022-01258-3

**Published:** 2022-01-25

**Authors:** Liping Hong, Mingfei Pan, Xiao Yang, Xiaoqian Xie, Kaixin Liu, Jingying Yang, Shan Wang, Shuo Wang

**Affiliations:** State Key Laboratory of Food Nutrition and Safety, Tianjin University of Science and Technology, Tianjin, 300457 China

**Keywords:** β-Lactoglobulin, Molecularly imprinted fluorescence sensor, Upconversion micro-particles, Metal–organic frameworks, Thermo-sensitive

## Abstract

In this study, a thermo-sensitive molecularly imprinted fluorescence sensor was developed for the specific detection of β-Lactoglobulin (β-LG) allergen in milk products. The metal–organic frameworks (MIL-100) with a high specific surface area was coated on the surface of upconversion micro-particles (UCMPs). As the core, an imprinted polymer layer allowing for swelling and shrinking with response to temperature was prepared, which exhibited high adsorption and mass transfer capabilities for β-LG allergen. The fluorescence intensity of UCMPs@MIL-100@MIP decreased linearly with the concentration of β-LG in the range of 0.1–0.8 mg mL^−1^, and the limit of detection was 0.043 mg mL^−1^. The imprinting factor reached 3.415, which indicated that excellent specificity of the UCMPs@MIL-100@MIP for β-LG allergen. In the analysis of β-LG allergen in actual milk samples, the proposed UCMPs@MIL-100@MIP fluorescence sensor produced reliable and accurate results (recovery: 86.0–98.4%, RSD: 2.8–6.8%), closely related to the results of standard HPLC method (correlation coefficient: 0.9949), indicating that its feasibility in the detection of β-LG allergen.

**Graphical Abstract:**

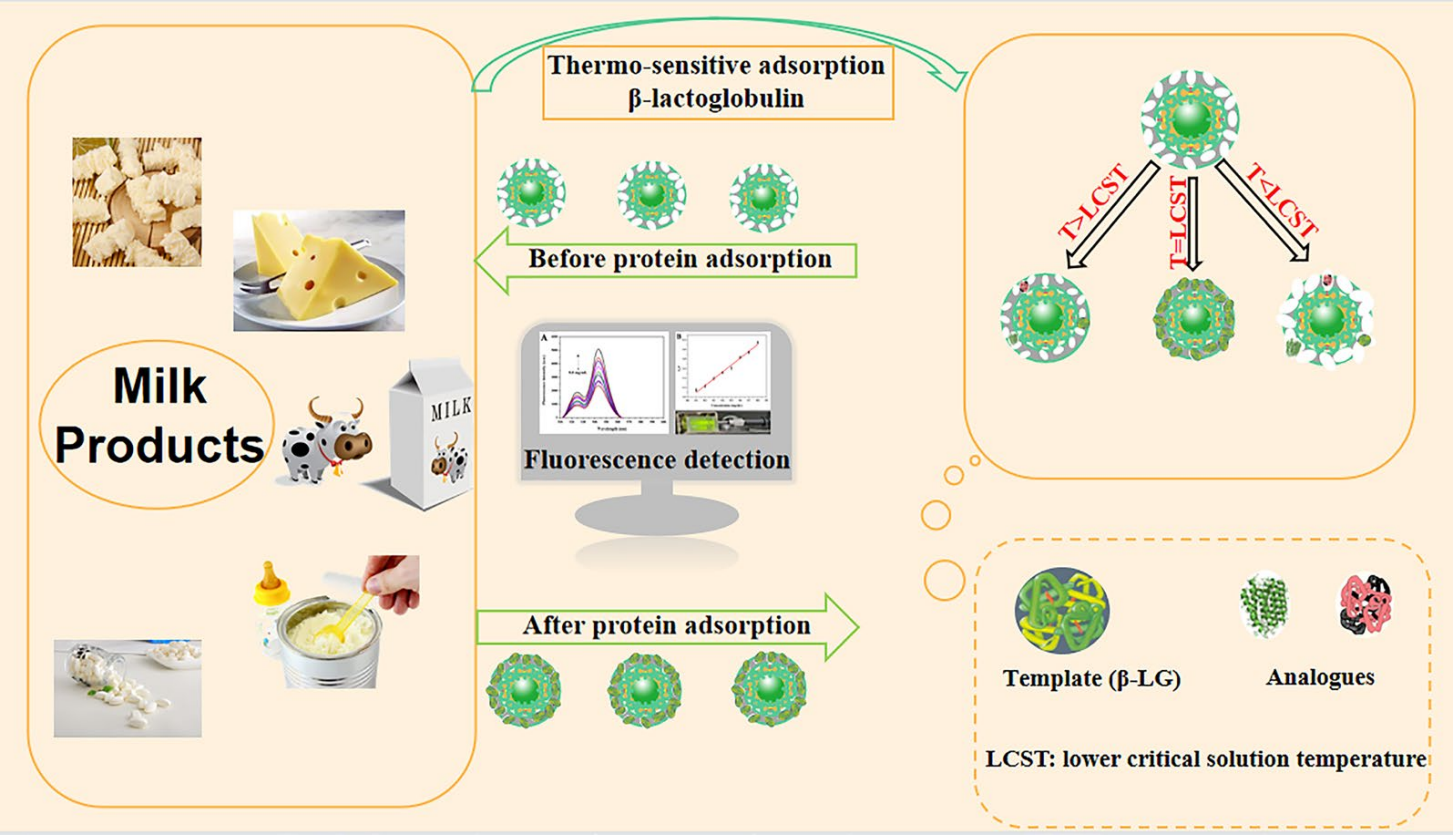

**Supplementary Information:**

The online version contains supplementary material available at 10.1186/s12951-022-01258-3.

## Introduction

Milk allergy seriously affects the life of people allergic to milk and has a relatively high incidence worldwide [1]. β-lactoglobulin (β-LG), belonging to the strong allergen lipocalin family, is one of the milk proteins that can cause strong allergic reactions such as rash, vomit, diarrhea, and abdominal cramps [2–4]. According to epidemiological survey results, approximately 82% of milk allergy patients are allergic to β-LG [5, 6]. However, milk products are also a special industry with great attention in the agriculture and animal husbandry industry chain. In the process of food processing, β-LG is also widely added to increase nutrition of foods, increasing the risk of exposure to β-LG in people with milk allergies [7–9]. Therefore, it is necessary to develop a convenient, accurate, effective and specific analytical method to monitor the content of β-LG in milk products, which beneficial to protect consumers from the threat of allergies. High-performance liquid chromatography (HPLC) is the standard strategy for quantitative analysis of β-LG. Due to the precision requirement of the instrument, this method usually requires strict sample pretreatment process and longer analysis time, resulting in high detection cost [10]. Enzyme-linked immunosorbent assay (ELISA) has been widely used in the detection of β-LG due to its high specificity and sensitivity. However, under the influence of various external conditions such as food processing, false positive and false negative results may occur [11]. Surface plasmon resonance and electrochemical sensors have the advantages of high sensitivity, strong anti-interference ability, and simple preparation in food analysis [6, 12]. However, due to the instability of protein macromolecules, the accuracy of such sensor devices is reduced, which limits their further applications. Therefore, it is of great value to design a synthetic, low-cost and highly specific bionic sensing system to detect β-LG.

Inspired by the "antigen and antibody binding", molecularly imprinted polymers (MIPs) have attracted much attention in the field of protein detection [13]. However, traditional MIPs have defects such as limited mass transfer capacity, poor structural rigidity, poor accessibility to target molecules and uncontrollable adsorption process, leading to a decrease in the adsorption capacity, adsorption rate and selectivity of MIPs to targets [14, 15]. Due to the characteristics of phase transition and structural transition under temperature stimulation, thermo-sensitive MIPs have remarkable adsorption reversibility and stability, showing great potential in the field of biomolecular recognition [16–18]. Combining this "smart material" with fast-responding and simple-to-operate fluorescence sensing technology is an effective strategy to improve the selectivity and specificity of sensors in β-LG analysis [19]. Nevertheless, traditional quantum dots fluorescent materials are highly toxic, and ultraviolet radiation will cause irreversible damage to biological samples such as proteins, which greatly limits their application [20, 21]. Therefore, it is necessary to synthesize a luminescent material that does not emit ultraviolet fluorescence.

Upconversion micro-particles (UCMPs) is a kind of fluorescent materials doped with lanthanide rare earth elements, which can emit high-energy visible light under the excitation of low-energy infrared or near-infrared light [22, 23]. UCMPs with low toxicity, good biocompatibility and low biological fluorescence background values have gained increasing attention in detection and biomarkers [24–26]. However, the adverse factors such as poor dispersion, small specific surface area and unstable luminescence limit the further application of single-component materials. Composite materials prepared by coating the UCMPs surface can solve these problems [27, 28]. Metal–organic frameworks (MOFs) is a kind of crystalline material with regular network topology formed by self-assembly of nitrogen or oxygen-containing organic ligands and metal centers [29–31]. Among various MOF materials, MIL-100 (Fe) has the characteristics of large specific surface area, uniform pore size distribution and adjustable pore structure, and has been widely used in gas storage, catalysis, separation, drug transportation and photoelectric sensing [32, 33].

Herein, a feasible and effective strategy was proposed in this study for the detection of β-LG in milk products. A core–shell composite with UCMPs as core and MIL-100 (Fe) as shell was prepared and used as a carrier to develop fluorescent thermo-sensitive MIP materials. This material combined the fluorescence characteristic of UCMPs, the high specific surface area of MIL-100 (Fe) and the high specificity recognition of thermo-sensitive MIP, and has a specific, rapid and stable fluorescence response to β-LG, which can be used for convenient and efficient analysis of β-LG allergen in milk products.

## Materials and methods

### Materials and chemicals

The proteins β-LG (pI 5.1–5.3, 90%), α-lactalbumin (ALa, pI 4.2–4.5, 85%), lactoferrin (Lf, pI 8.0, 95%), and casein (Cas, pI 4.8, > 95%) were obtained from Yuanye Biotech Co. Ltd. (Nanjing, China). Y(CH_3_COO)_3_·H_2_O (99.9%), Yb(CH_3_COO)_3_·4H_2_O (99.9%), Er(CH_3_COO)_3_·4H_2_O (99.9%), *N,N*-methylenebisacrylamide (MBA), oleic acid (OA, 90%), ethylene glycol (EG, > 99%), *N,N,N',N'-*tetramethylethylenediamine (TEMED, 99%), polyacrylic acid (PAA), and *N*-isopropylacrylamide (NIPAM), were provided by Macklin Biotech Co. Ltd. (Shanghai, China). Trisodium citrate dihydrate (TSC, 99.0%), ammonium persulfate (APS), trimesic acid (H_3_BTC), NaCl (99.5%), NH_4_F and FeCl_3_·6H_2_O were purchased from Aladdin Reagent Co., Ltd. (Shanghai, China). The reagents anhydrous ethanol, acetic acid, trifluoroacetic acid (TFA, > 99.9%), and acetonitrile at least of analytical grade were acquired from National Medicine Group Chemical Reagent Co., Ltd. (Shanghai, China). Deionized water (18.2 MΩ cm) was obtained by a Water Pro water purification system (Labconco, Kansas City, USA).

### Instrumentation

The UV absorbance at a wavelength of 280 nm was recorded on the Evolution 300 UV–Vis spectrophotometer (Thermo, USA). Infrared spectra were obtained using a Fourier transform infrared spectrometer (Nicolet iS 50, Thermo, USA). Fluorescence measurements were performed on an F-7100 fluorescence spectrophotometer (Techcomp, Shanghai, China) equipped with a 980 nm external exciter (2 W, Shanghai Feibo Laser Technology Co., China) as the light source. Transmission and scanning electron microscopy (TEM and SEM) images were obtained from a Talos G2 200X and Apreo electronic microscope (Thermo, USA), respectively. X-ray powder diffraction (XRD) patterns were recorded on a Malvern Panalytical apparatus at a scanning rate of 1° min^−1^ in the 2θ range from 5° to 80° (Shanghai, China). Energy dispersive X-ray photoelectron spectroscopy (XPS) patterns were measured on ARL QUANT'X Energy dispersive X-ray spectrometer (Thermo, USA). Nitrogen adsorption/desorption analysis was performed on a 3H-2000PS apparatus (Beijing, China) with a bath temperature of 77 K. Millipore ultrafine filters from Merck Company (Germany) and a centrifuge machine from Eppendorf (Germany) were used in the pre-treatment of milk products. A HPLC system equipped an SPD-20A detector from Shimadzu Corporation (Japan) was applied to verify the results of β-LG measurement in milk products.

### Synthesis and modification of UCMPs

Hexagonal NaYF_4_: Yb^3+^, Er^3+^ UCMPs were synthesized refer to the previous work with a little modification [34]. At room temperature, 8 mmol of TSC were dissolved in 20.0 mL of H_2_O in a 100.0 mL reaction flask. Another 10.0 mL of homogeneous aqueous solution containing 1 mmol of rare earth salt (Y^3+^: Yb^3+^: Er^3+^  = 0.78: 0.2: 0.02) was added and the mixture was stirred magnetically for 10 min. Then, 10.0 mL of a mixed solution containing 337.0 mg (5.76 mmol) of NaCl and 444.0 mg (12 mmol) of NH_4_F was added into the flask dropwise, gradually forming a milky solution. Subsequently, 20.0 mL of OA and 10.0 mL of EG were slowly added into the milky solution successively. After stirring for another 1.5 h, the mixture was transferred into a 100.0 mL stainless-steel autoclave for solvothermal reaction in Muffle furnace, sealed and kept at 180 °C for 6 h. After the autoclave was cooled to room temperature, the white products (UCMPs-OA) were obtained by centrifugation, fully washed with ethanol and dried at 60 °C.

Furthermore, a ligand exchanging process of OA and PAA was carried out to ensure that the fluorescence probe UCMPs could be applied effectively for protein detection. 100.0 mg of UCMPs-OA product were washed thoroughly with ultrasound in 10.0 mL of ethanol (adjusted with HCl) at pH 1.0, and then dispersed in 10 mL of ethanol solution after centrifuging. The dispersion was dropped into ethanol solution containing 30.0 mg of PAA and stirred magnetically at room temperature for 12 h. After removing excess PAA by centrifugation and ethanol washing, the product UCMPs-PAA was dried at 60 ℃ for 10 h.

### Synthesis of UCMPs@MIL-100

The detailed synthesis process of UCMPs@MIL-100 was as follows. 100.0 mg of UCMPs-PAA product was mixed with 30.0 mL of ethanol in a reaction vessel thoroughly. 13.5 mg (0.05 mmol) of FeCl_3_·6H_2_O was added into the vessel and the mixture was magnetically stirred for 30 min at room temperature to allow a stable connection between Fe^3+^ and UCMPs-PAA. Subsequently, 10.0 mL of an ethanol solution containing 10.5 mg (0.05 mmol) of H_3_BTC was added and the mixture was stirred and heated in a water bath at 40 ℃ for 40 min, and the solid product was collected by centrifugation. The same procedure was repeated 10 times to coat MIL-100 uniformly on the surface of UCMPs. The final product was washed alternately with water and ethanol and dried at 60 ℃ for 10 h in a vacuum drying oven to obtain UCMPs@MIL-100 light yellow powder.

### Synthesis of UCMPs@MIL-100@MIP and UCMPs@MIL-100@NIP

Accurately weighted β-LG (10.0 mg), and the prepared UCMPs@MIL-100 (50.0 mg) were dispersed in 10.0 mL of water in a 25.0 mL round-bottom flask and shaken for 10 min. Subsequently, 79.0 mg (0.7 mmol) of NIPAM and 162.0 mg (1.05 mmol) of MBA were added, and shaken at room temperature for 1 h. After adding 10.0 mg of APS and 100.0 μL of TEMED (5%, v/v) and nitrogen bubbling for 10 min to remove oxygen, the polymerization reaction was carried out at 32 °C for 24 h. After the polymerization was completed, the template β-LG was alternately eluted with 0.5 mol L^−1^ NaCl and acetic acid (6.0 vt%) at 20 ℃ for several times, and until no β-LG was detected by UV–vis spectrophotometer. The UCMPs@MIL-100@NIP was prepared adopting the same procedure except the addition of β-LG.

### Fluorescence measurement and thermo-sensitive adsorption experiments

All fluorescence analysis was performed using an F-7100 fluorescence spectrometer equipped with a 980 nm external exciter, which recorded emission wavelengths in the 400–700 nm range. 2.0 mg of UCMPs@MIL-100@MIP was mixed with 2.0 mL of β-LG solution with a certain concentration in a 5.0 mL centrifuge tube. After shaking for 1 h, the fluorescence intensity of mixture was measured quickly (λ = 544 nm).

The adsorption performance of UCMPs@MIL-100@MIP and NIP at different temperature was investigated by mixing 2.0 mg of the prepared UCMPs@MIL-100@MIP or NIP with 2.0 mL of β-LG solution (0.2 mg mL^−1^) and shaken at different temperatures (20, 32, 44 ℃) for 1 h. After centrifugation at 5000 rpm, the concentration of β-LG in supernatant was measured by UV–vis spectrophotometer.

### Sample preparation and validation of method

In this study, raw milk and infant formula were selected as actual samples to study the application capability of the prepared UCMPs@MIL-100@MIP fluorescence sensor. Accurately weighted raw milk (5.0 mL) or infant formula (2.0 g), were placed into a 25.0 mL volumetric flask, respectively. After reaching a constant volume and adjusting pH to 4.6 using acetic acid, the mixture was allowed to stand for 1 h and centrifuged for 10 min (5000 rpm). The collected supernatant was filtered through a 0.22 µm filter, immediately afterwards, the raw milk supernatant was diluted 2 times, and analyzed using the prepared fluorescence sensor. The resulting fluorescence intensity was used to calculate the amount of β-LG. The HPLC separation was achieved on a Hypersil GOLD C_8_ column (4.6 × 250 mm, 3.5 µm), and H_2_O (0.1% TFA) (A) and acetonitrile (0.1% TFA) (B) were used as the mobile phase. The gradient conditions at a flow rate of 1.0 mL min^−1^ were as follows: A, from 70 to 50% (0–15 min); A, from 50 to 70% (15—17 min). The detection wavelength and injection volume were 280 nm and 10.0 µL, respectively. The results of β-LG analysis from the prepared fluorescence sensor and HPLC were compared, and the correlation coefficient (R^2^) was calculated.

## Results and discussion

### Preparation of UCMPs@MIL-100@MIP

**Scheme 1:**
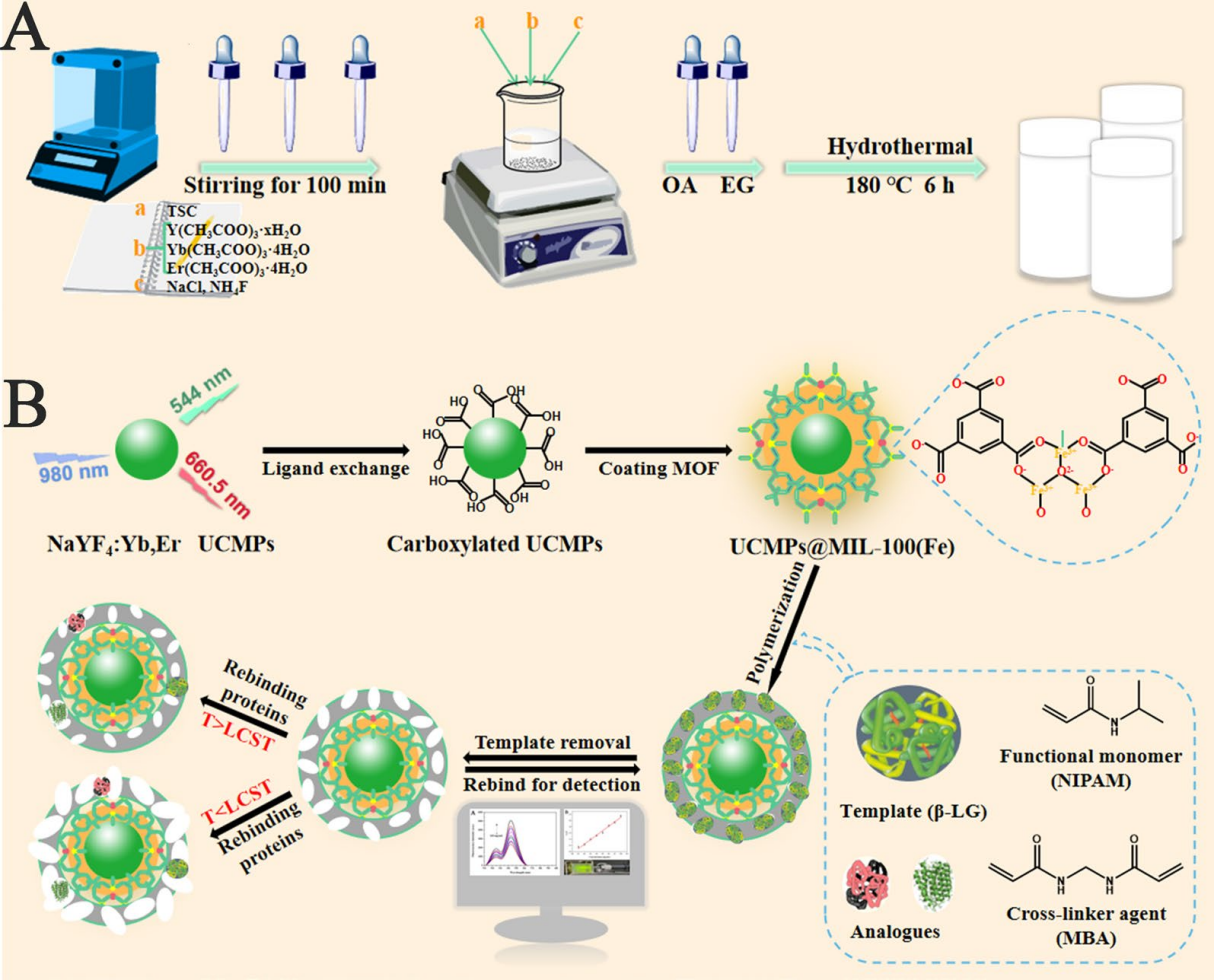
**A** The synthesized process of UCMPs; **B** the detailed preparation procedure of UCMPs@MIL-100@MIP

This study combined the fluorescence characteristics of UCMPs, the high specific surface area of MIL-100 material, and the specific recognition capability of thermo-sensitive MIP to develop a fluorescence strategy for the effective detection of β-LG. Scheme 1A shows the synthesis process of NaYF_4_: Yb^3+^, Er^3+^ UCMPs with green fluorescence by solvothermal method. Through the ligand exchanging process between OA and PAA, the synthetic UCMPs were transformed into hydrophilic UCMPs (Scheme 1B). Due to the interaction between Fe^3+^ and the carboxyl groups of UCMPs and H_3_BTC, MIL-100 framework was wrapped around the hydrophilic UCMPs. The template protein β-LG was immobilized onto the surface of UCMPs@MIL-100 by non-covalent interaction, and the MIP layer was prepared by polymerizing NIPAM and MBA in aqueous solution. After eluting the template protein β-LG, UCMPs@MIL-100@MIP with specific recognition sites was obtained.

Under the action of thermo-sensitive monomer NIPAM, the adsorption and desorption performance of UCMPs@MIL-100@MIP could be achieved by controlling the external temperature. When the temperature was lower than lower critical solution temperature (LCST), NIPAM exhibited hydrophilicity, and the expanded imprinting cavities had no complementary affinity to the template protein functionally and spatially. When the temperature was higher than the LCST, the NIPAM was in a hydrophobic state, and the imprinting cavities shrunk. At this time, although the imprinted cavities were not complementary to the template protein, the hydrophobic interaction played a dominant role and the template would also be captured.

### Characterization of UCMPs@MIL-100@MIP

#### SEM and TEM analysis

**Fig. 1:**
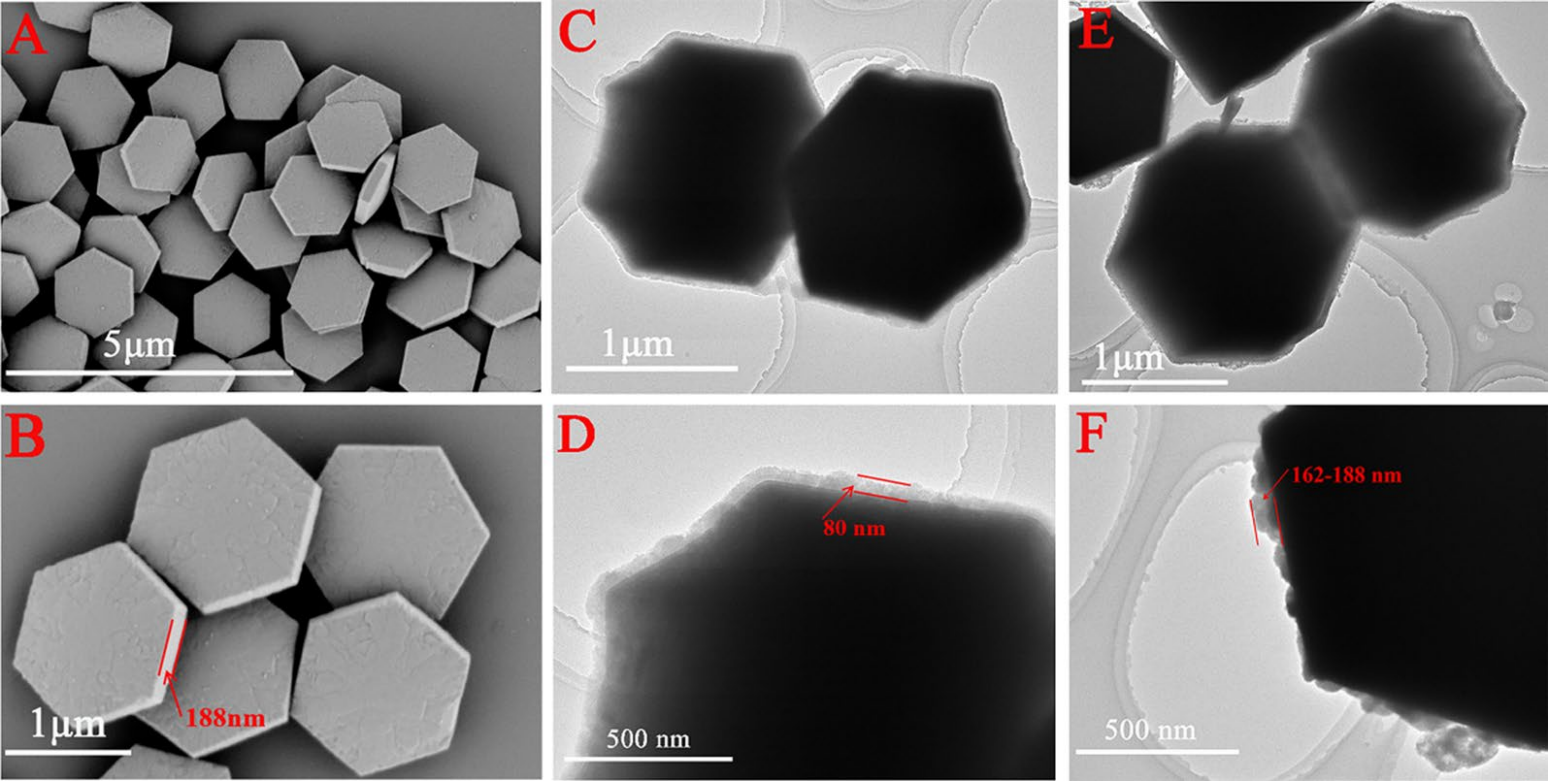
SEM images of UCMPs (**A**, **B**), TEM images of UCMPs@MIL-100 (**C**, **D**) and UCMPs@MIL-100@MIP (**E**, **F**)

SEM and TEM images were used to observe the surface morphology and size of the synthetic UCMPs, UCMPs@MIL-100 and UCMPs@MIL-100@MIP (Fig. 1). Evidently, the bare UCMPs showed regular hexagonal with a particle size of about 1.5 μm and a thickness of about 188 nm (Fig. 1A, B). After the surface of UCMPs was coated with MIL-100, the size of the UCMPs@MIL-100 increased significantly and the coating thickness of MIL-100 film was about 80 nm (Fig. 1C, D). Accompanied by the growth of MIP upon the surface of UCMPs@MIL-100, the size of the coating layer has expanded to 162–188 nm (Fig. 1E, F), and the shape was irregular, confirming the successful preparation of UCMPs@MIL-100@MIP.

#### FT-IR spectra analysis

**Fig. 2:**
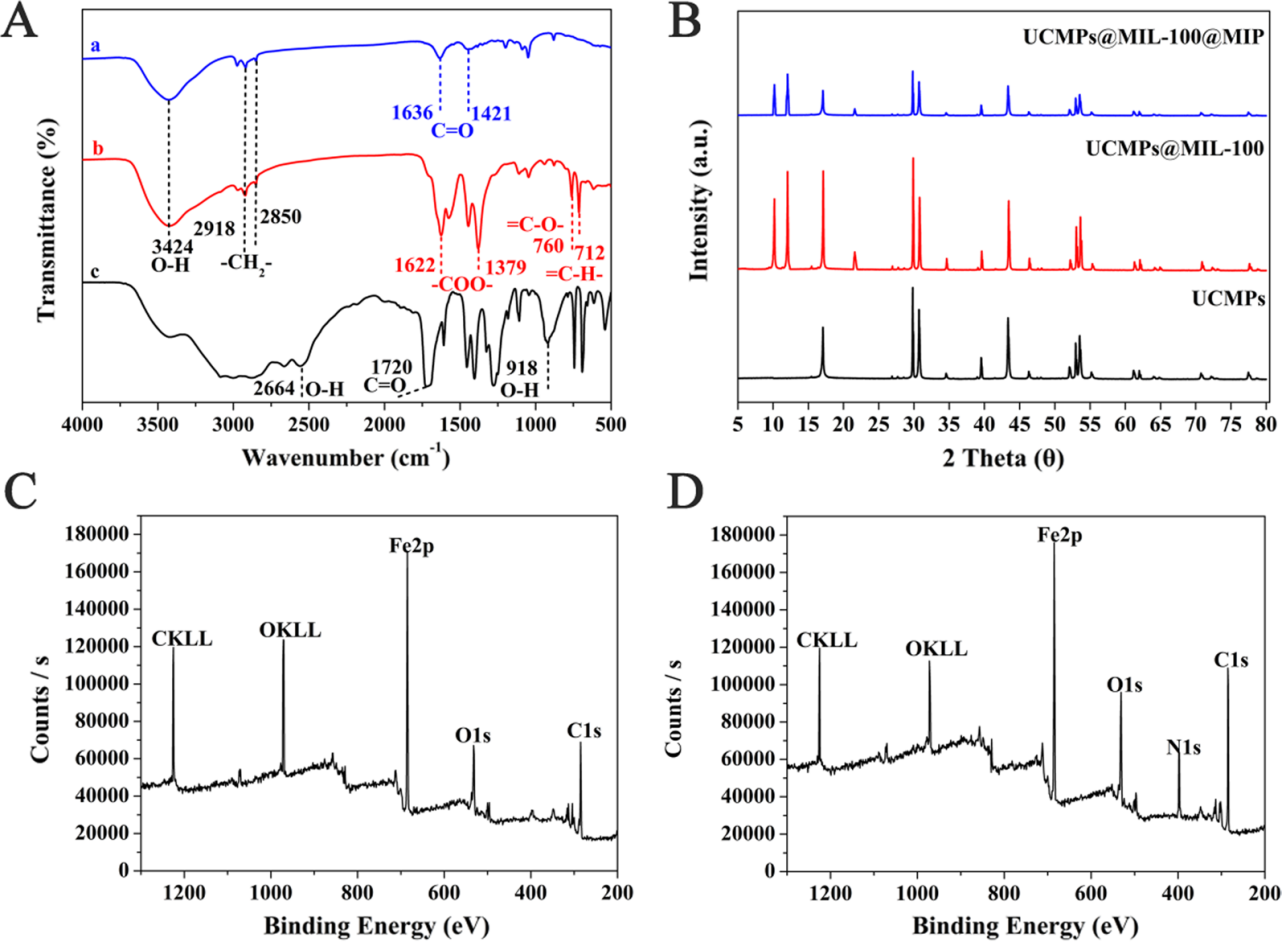
**A** FT-IR spectra of UCMPs-PAA (a), UCMPs@MIL-100 (b) and H_3_BTC (**c**); **B** X-ray diffraction patterns of the obtained UCMPs (black), UCMPs@MIL-100 (red) and UCMPs@MIL-100@MIP (blue); XPS spectra for **C** UCMPs@MIL-100 and **D** UCMPs@MIL-100@MIP

The UCMPs-PAA, UCMPs@MIL-100 and H_3_BTC were characterized by FT-IR spectroscopy. At 2850 and 2918 cm^−1^, there were symmetrical stretching vibration peaks and anti-symmetric stretching vibration peaks, respectively, which suggested the stretching vibration of the –CH_2_– on PAA (Fig. 2A(a)). The characteristic peaks at 1421 and 1636 cm^−1^ were attributed to the stretching vibration of –COOH groups. These results indicated that UCMPs were successfully modified with PAA ligands.

The characteristic peaks at 2664, 1720 and 918 cm^−1^ in Fig. 2A(c) correspond to the stretching vibration of the O–H, C=O, and the bending vibration of the O–H in H_3_BTC, respectively. In the FT-IR spectra of UCMPs@MIL-100 (Fig. 2A(b)), the above three main characteristic peaks disappeared, and two significant absorption peaks appeared at 1622 and 1379 cm^−1^, which related to the symmetric and asymmetric stretching vibrations of ionized –COO^−^, respectively. This indicated that the –COOH groups of H_3_BTC dissociated into –COO^−^ anions and formed coordination bonds with Fe^3+^. In addition, the fingerprint peaks derived from the vibration of the benzene ring were observed at 760 and 712 cm^−1^. These FT-IR spectra results were in full agreement with the step-by-step assembly process of MIL-100, indicating that the surface of UCMPs has been successfully coated by MIL-100 framework.

### XRD and XPS analysis

Figure 2B has illustrated the crystal phase structure of UCMPs, UCMPs@MIL-100 and UCMPs@MIL-100@MIP determined by XRD. In the XRD diagram of UCMPs@MIL-100, UCMPs diffraction peaks were observed to be well preserved, and part of the characteristic peaks were consistent with those of MIL-100 previously reported, suggesting that the composite material was composed of UCMP_S_ and MIL-100. In addition, the peak intensity of UCMPs@MIL-100@MIP was significantly reduced compared with that UCMPs@MIL-100. The peak intensity reflected the crystallization of the material, so it was speculated that the reason for the weakening was the formation of the MIP film on the surface. These results confirmed the successful preparation of UCMPs@MIL-100@MIP, which was consistent with the results of above characterization results.

XPS exhibited the corresponding elements of UCMPs@MIL-100 and UCMPs@MIL-100@MIP. In Fig. 2C, the primary signals of C1s at 284.81 and 288.6 eV, O1s at 531.71 eV and Fe2p at 712.09 eV can be clearly observed, indicating that the MIL-100 has been successfully coated on the surface of the UCMPs. Compared with Fig. 2C, the N1s peak was clearly observed at 397.02 eV in Fig. 2D. The N source mainly derived from the N elements carried by β-LG, NIPAM, and MBA during the formation of the MIP layer, which confirmed the MIP was successfully coated on the surface of UCMPs@MIL-100.

### Fluorescence quenching mechanism of β-LG to UCMPs@MIL-100@MIP

In the study, the prepared UCMPs and UCMPs@MIL-100 materials (1.0 mg) were dispersed in 2.0 mL of water to investigate the fluorescence properties. As shown in (Additional file 1: Fig. S1A), under the excitation of the external 980 nm laser, UCMPs and UCMPs@MIL-100 appeared green fluorescence emission peaks at 529 nm and 544 nm, respectively, which was due to the transition of Er^3+^ between the ^2^H_11/2_ → ^4^I1_5/2_ and ^4^S_3/2_ → ^4^I_15/2_ energy levels. Therefore, the maximum emission peak at 544 nm was selected as a marker to evaluate the fluorescence characteristics of the synthesized materials. Due to the fluorescence quenching of UCMPs caused by MIL-100 coating, the fluorescence intensity of UCMPs@MIL-100 was significantly lower than that of UCMPs. These results preliminarily proved the successful synthesis of UCMPs@MIL-100 composites.

As shown in (Additional file 1: Fig. S1B), compared with UCMPs@MIL-100@NIP (a), UCMPs@MIL-100@MIP without removing β-LG has lower fluorescence intensity (c). After β-LG was removed, the fluorescence intensity of UCMPs@MIL-100@MIP (b) was significantly enhanced, almost close to that of NIP, which verified the quenching effect of β-LG on the fluorescence of UCMPs. Studies have proved that the main mechanism that caused fluorescence quenching were fluorescence resonance energy transfer (FRET) and photoinduced electron transfer (PET) in recent years. Nevertheless, FRET occurred when the excitation band of the fluorescent receptors and the emission band of the donors overlapped in the analysis system. According to (Additional file 1: Fig. S1C), the absorption peak of β-LG at 280 nm did not overlap with the emission peak of the fluorophore. Therefore, the fluorescence quenching effect was probably caused by electron transfer.

### Thermo-sensitive property of the UCMPs@MIL-100@MIP

**Fig. 3:**
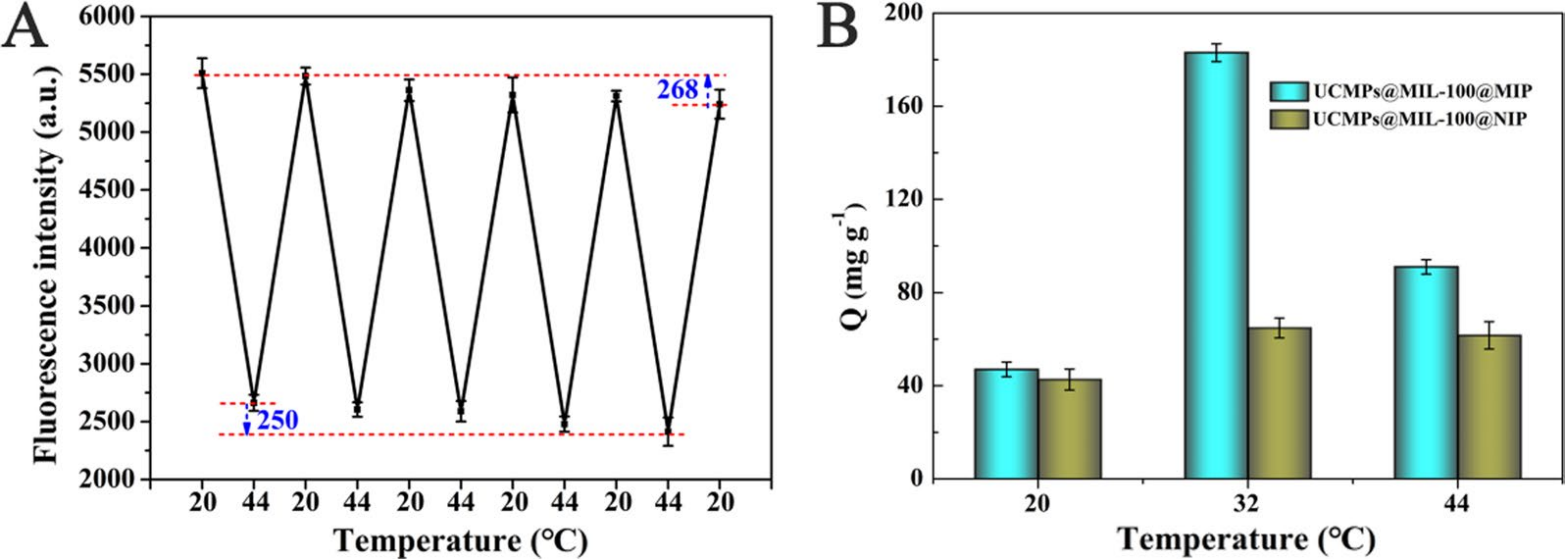
**A** Changes of the fluorescence intensity of UCMPs@MIL-100@MIP for β-LG at 20 ℃ and 44 ℃; and **B** the adsorption capacity of the UCMPs@MIL-100@MIP for β-LG at 20 ℃, 32 ℃ and 44 ℃

It was well known that NIPAM-based polymers exhibited both hydrophilic and hydrophobic state at different temperatures, simultaneously, the volume of polymers would change with the external temperature. As a result, the influence of temperature on the adsorption capacity of the prepared UCMPs@MIL-100@MIP was investigated. (Additional file 1: Fig. S2) shows the fluorescence intensity of the UCMPs@MIL-100@MIP without adding the template protein β-LG at 20 °C and 44 °C. Figure 3A shows the fluorescence intensity of the UCMPs@MIL-100@MIP in adsorption to β-LG at 20 °C and 44 °C, indicating that a significant temperature dependence of their interactions. After five cycles, the fluorescence intensity of the thermo-sensitive UCMPs@MIL-100@MIP was almost unchanged, which indicated its good fluorescence anti-attenuation ability. These results demonstrated that the UCMPs@MIL-100@MIP could achieve β-LG adsorption and desorption by controlling the external temperature, laying a foundation for its repeatable use in fluorescence sensing.

Furthermore, the adsorption capacity (*Q*, mg g^−1^) of UCMPs@MIL-100@MIP and NIP for β-LG was calculated by the following equation.

1 $$\mathrm{Q}=({\mathrm{C}}_{0}-\mathrm{C})*\mathrm{V}/\mathrm{m}$$

In which, *C*_0_ and *C* represents the initial and residual concentration of β-LG, mg mL^−1^, respectively; *V* is the volume of β-LG solution, mL; and *m* represents the mass of MIP or NIP, g.

As shown in Fig. 3B, the adsorption capacity of the prepared UCMPs@MIL-100@MIP for β-LG reached 183.0 mg g^−1^ at 32 °C, which was significantly higher than that at 20 °C (47.0 mg g^−1^) and 44 °C (90.9 mg g^−1^). This was because the shape and size of the imprinted sites or cavities formed in the polymer were complementary to β-LG at 32 °C. At 20 °C, the NIPAM monomer was hydrophilic and formed a large number of hydrogen bonds in water, which enlarged the imprinted cavities of the polymer and resulted in most β-LG molecules entering and leaving unrecognized. At high temperature (44 °C), the hydrogen bonds formed by NIPAM were destroyed and the hydrophobic action dominated, leading to shrinkage of the polymer cavities in the aqueous phase. This hydrophobic effect also led to a significant increase in non-specific adsorption, making its adsorption capacity higher than 20 °C, which was consistent with the study of Zhou et al. [35]. In addition, UCMPs@MIL-100 material had a larger specific surface area than traditional carrier materials, reaching 637.38 m^2^ g^−1^, measured by nitrogen adsorption/desorption isotherm. This was also one important reason why UCMPs@MIL-100@MIP had stronger adsorption performance for the target protein.

### Optimization of UCMPs@MIL-100@MIP preparation conditions

In the study, the amount of UCMPs@MIL-100, the molar ratio of functional monomer and cross-linker, and the adsorption environment (pH) were investigated to obtain the optimal adsorption performance of UCMPs@MIL-100@MIP. The variable control method was adopted, and the imprinting factor (*IF*) was used as the final evaluation index. The amount of UCMPs@MIL-100 prepared as the fluorescence source is closely related to the sensitivity of the constructed fluorescence sensor. (Additional file 1: Fig. S3A) shows the fluorescence responses of MIP and NIP prepared using different amounts of UCMPs@MIL-100. It can be observed that the addition amount of UCMPs@MIL-100 significantly affected the fluorescence response of the prepared MIP and NIP. When the addition amount was 50 mg, the maximum value of *IF* was 2.465. The cross-linker can form a stable rigid structure, which was conducive to the curing of the functional monomer in the polymerization layer, and then forming cavities or binding sites that match the template molecules. When the cross-linker was insufficient, the network structure of imprinted layer cannot be well connected, which affected the adsorption of β-LG molecule by MIP. Nevertheless, superfluous cross-linker will increase the thickness of the imprinted layer, resulting in mass transfer barrier, which will not only affect the mass transfer speed of β-LG in the imprinted layer, but also hinder its interaction with the fluorescence source UCMPs@MIL-100, thus decreasing the detection sensitivity of the fluorescence sensor. By comparing the fluorescence response of UCMPs@MIL-100@MIP and NIP prepared under different ratios of functional monomers and cross-linker (Additional file 1: Fig. S3B), the *IF* reached the maximum value (2.790) at the molar ratio of 2/3, which was chosen for further experiments. In addition, when the adsorption environment pH was 7.4 (Additional file 1: Fig. S3C), the best *IF* value of 3.208 was obtained. This was because when the pH of the solution was below 7.4, β-LG has less positive charge on its surface, while the alkalinity of UCMPs@MIL-100@MIP and the solution environment was relatively weak. When pH = 7.4, the surface positive charge of β-LG increased, and the alkalinity of UCMPs@MIL-100@MIP was stronger than that of solution system, indicating that UCMPs@MIL-100@MIP played an important role in the recognition and retention of β-LG. With the continuous increase of pH value, the affinity of the solution system to β-LG gradually dominated, leading to gradually lost the recognition ability of UCMPs@MIL-100@MIP.

### Fluorescence response of UCMPs@MIL-100@MIP to β-LG

**Fig. 4:**
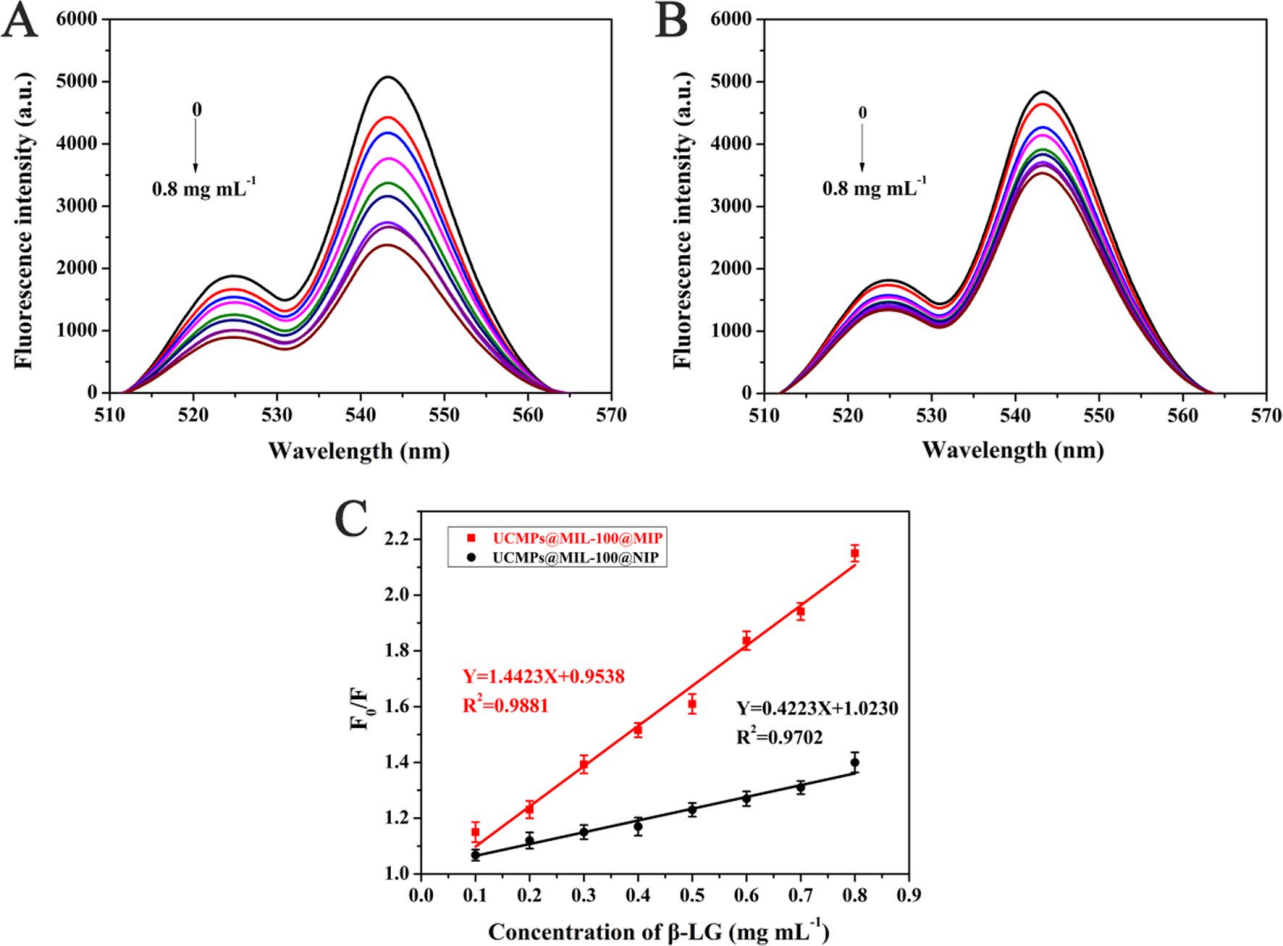
Fluorescence spectra of UCMPs@MIL-100@MIP (**A**) and NIP (**B**) at different concentrations of β-LG, and (**C**) Stern–Volmer plots from UCMPs@MIL-100@MIP (red) and NIP (black) with β-LG

In this work, the fluorescence response of prepared UCMPs@MIL-100@MIP and NIP to different concentrations of β-LG allergen were evaluated. As shown in Fig. 4C, the fluorescence response value (*F*_0_/*F*) of UCMPs@MIL-100@MIP was significantly correlated with the concentration of β-LG in the range of 0.1—0.8 mg mL^−1^, in line with the following Stern–Volmer equation.

2 $${F}_{0}/F={K}_{\mathrm{SV}}C+1$$

In which, *F*_0_ and* F* respectively represented the fluorescence intensity before and after the adsorption of β-LG, *K*_SV_ was the quenching constant, and *C* represented the β-LG concentration (mg mL^−1^). In addition, the limit of detection (LOD) can be calculated according to the following formula:

3 $${C}_{L}=3{S}_{b}/M$$

In which, *C*_L_ represented the LOD (mg mL^−1^), *S*_b_ was the blank standard deviation, and *M* represented the slope of the standard curve.

The fluorescence quenching equation of UCMPs@MIL-100@MIP was *F*_0_*/F* = 1.4423 *C* + 0.9538 with *R*^2^ of 0.9881, and the LOD was calculated as 0.043 mg mL^−1^. Compared with the fluorescence spectra of NIP at the same β-LG concentration, the quenching degree of UCMPs@MIL-100@MIP was obviously higher (Fig. 4A, B). This was because more binding cavities or recognition sites matching the size and shape of β-LG protein were formed in the imprinted layer. By comparing the slope of the fluorescence quenching equation (Fig. 4C), the *IF* was calculated as 3.415, indicating that the prepared UCMPs@MIL-100@MIP had good selectivity and specificity for β-LG.

### Kinetics evaluation of UCMPs@MIL-100@MIP

To evaluate the kinetic properties of the prepared UCMPs@MIL-100@MIP and NIP, the equilibrium binding analysis was performed at a β-LG concentration of 0.4 mg mL^−1^. As can be seen from (Additional file 1: Fig. S4), the adsorption rate of UCMPs@MIL-100@MIP increased within 30 min and almost reached the adsorption equilibrium within 60 min. In the same period of adsorption, the *F*_0_/*F* change of UCMPs@MIL-100@MIP for β-LG was more significant than that of UCMPs@MIL-100@NIP. This was because UCMPs@MIL-100@MIP generates imprinted sites with respect to β-LG during the preparation and has specific and non-specific binding during the adsorption process. However, UCMPs@MIL-100@NIP only existed non-specific adsorption. In addition, these results also indicated that the introduction of MIL-100 material not only increased the number of β-LG specific recognition sites in imprinting system, but also arranged the specific recognition sites in order, which was beneficial to the rapid binding of β-LG. This verified the merits of this work in improving the adsorption capacity and efficiency of the molecular imprinting system.

### Selectivity study

**Fig. 5:**
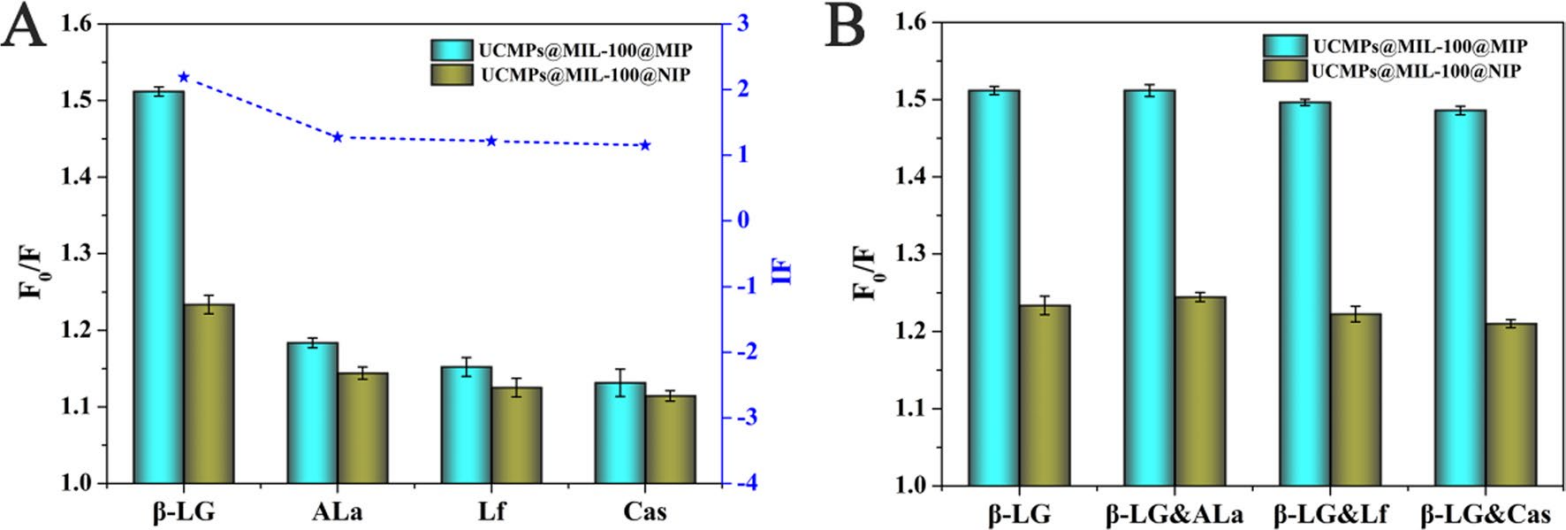
**A** Fluorescence responses of UCMPs@MIL-100@MIP and NIP to β-LG and the competing proteins; **B** Fluorescence responses of UCMPs@MIL-100@MIP in the mixed system of β-LG and interfering proteins

The selectivity of UCMPs@MIL-100@MIP was evaluated using ALa, Lf, and Cas as competitive proteins at 0.4 mg mL^−1^ concentration. As illustrated in Fig. 5A, it was clearly observed that the *F*_0_/*F* of UCMPs@MIL-100@MIP for β-LG changes more significantly than for ALa, Lf, and Cas. However, there was no significant difference in *F*_0_/*F* of UCMPs@MIL-100@NIP for the selected proteins. The *IF* was calculated as 2.19, 1.27, 1.21, and 1.15, respectively. This was because the specific cavities or recognition sites complementary to the size, shape, and functional groups of β-LG protein were formed during the preparation of UCMPs@MIL-100@MIP. However, due to the lack of template protein β-LG, UCMPs@MIL-100@NIP only formed non-specific adsorption sites, resulting in a small amount of target protein can be physically adsorbed.

Equal amounts of the interfering proteins were added into the β-LG solution (0.4 mg mL^−1^) to further investigate the anti-interference ability of the prepared UCMPs@MIL-100@MIP. As shown in Fig. 5B, the fluorescence response of UCMPs@MIL-100@MIP showed no significant changes in the three-protein mixed system compared to β-LG, indicating that its specific recognition ability for β-LG was not affected by the interfering proteins. UCMPs@MIL-100@NIP obtained a higher fluorescence response in mixed protein systems than each single interfering protein. These results indicated that the prepared UCMPs@MIL-100@MIP had significant specificity for β-LG and could be applied under the hindrance of the interferents in complex samples.

### Sample analysis and method validation

To evaluate the application capability of the prepared fluorescence sensor for analyzing β-LG in actual samples, raw milk and infant formula were selected and spiked with β-LG at three levels (0.1, 0.2, and 0.4 mg mL^−1^). After simple sample treatment, the β-LG content of the resulting extracts was measured using the prepared fluorescence sensor and validated by standard HPLC method. Table 1 illustrates the detection results of β-LG content obtained. The data listed was from a tenfold dilution of raw milk extract and a fivefold dilution of infant formula milk powder extract.

**Table 1:** Results of β-LG detection in milk products using the prepared fluorescence sensor and HPLC method

Samples	Initial concentration (mg mL^−1^)	Spiked levels (mg mL^−1^)	The prepared fluorescence sensor		HPLC	
			Found (mg mL^−1^)	Recovery (%, Mean ± SD, n = 3)	Found (mg mL^−1^)	Recovery (%, Mean)
Raw milk	0.34	0.10	0.42	93.8 ± 4.1	0.42	93.4
		0.20	0.50	87.9 ± 3.0	0.51	90.0
		0.40	0.74	98.4 ± 4.3	0.73	96.1
Infant formula	0.25	0.10	0.33	90.2 ± 2.5	0.32	86.5
		0.20	0.41	86.0 ± 5.8	0.42	89.8
		0.40	0.63	92.6 ± 4.3	0.64	94.5

Obviously, at all concentrations tested, the β-LG content detected by the prepared fluorescence sensor was similar to those obtained by HPLC, with a correlation coefficient achieving 0.9949 (Additional file 1: Fig. S5). This meant that the fluorescence sensor could be used for reliable and accurate analysis of β-LG. A comparison of the results of the reported strategies for β-LG analysis in various matrices was provided in Table 2, highlighting the merits of the prepared UCMPs@MIL-100@MIP fluorescence sensor.

**Table 2:** Comparison of the merits of the reported assays for β-LG detection in sample matrices

Methods	Matrices	Linear range (µg mL^−1^)	LOD (µg mL^−1^)	Required time	Reuse cycles	References
Ultra-HPLC	Ultra-high temperature treated milk	100–400	7.0	3 min	–	[10]
Sandwich ELISA	Defatted milk, yoghurt, and candy	0.03125–8	0.0196	> 20 min	Once	[11]
Surface plasmon resonance sensor	Final rinse water samples	0.49–1.0	0.168	< 1 min	–	[12]
Electrochemical method	Whey protein powders	53–11,160	27	15 min	Once	[6]
HPLC-bulk MIP	Fresh milk, pasteurized milk and powder milk	200–1400	70	> 2 h	At least 6	–
UCMPs@MIL-100@MIP fluorescence sensor	Raw milk and infant formula	100–800	43	1 h	At least 5	This work

## Conclusions

In this study, the fluorescence characteristic of UCMPs, the high specific surface area of MIL-100 and the high selectivity of molecular imprinting technology were effectively combined to prepare a thermo-sensitive molecular imprinted fluorescence sensor for the detection of β-LG allergen. The core–shell UCMPs@MIL-100@MIP material had good adsorption and recognition ability for β-LG allergen and can be controlled by the temperature of the adsorption system due to its thermo-sensitive effect. The prepared fluorescence sensor can accurately determine β-LG allergen with a concentration as low as 0.043 mg mL^−1^ in milk products, which had a broad application prospect in food safety, especially in the controllable detection of food allergens.

## Supplementary Information

**Additional file 1: Fig. S1.** (A) Fluorescence spectra of UCMPs (a) and UCMPs@MIL-100 (b); (B) fluorescence spectra of UCMPs@MIL-100@NIP (a), UCMPs@MIL-100@MIP before (b) and after (c) extraction; (C) UV–Vis spectra of β-LG (a) and fluorescence spectra of UCMPs@MIL-100@MIP (b); **Fig. S2.** Fluorescent spectra of UCMPs@MIL-100@MIP without protein in 20 ℃ and 44 ℃; **Fig. S3.** (A) Optimization of UCMPs@MIL-100 dosage, (B) addition ratio, and (C) the pH of the adsorption system; **Fig. S4.** Adsorption kinetics of UCMPs@MIL-100@MIP and UCMPs@MIL-100@NIP to β-LG; **Fig. S5.** Correlation curve of the results between standard HPLC and the prepared fluorescence sensor.
