# Supplementary material for: A UCMPs@MIL-100 based thermo-sensitive molecularly imprinted fluorescence sensor for effective detection of β-lactoglobulin allergen in milk products
Source: J Nanobiotechnology. 2022 Jan 25;20:51. doi: 10.1186/s12951-022-01258-3 (PMC8787952; doi:10.1186/s12951-022-01258-3)
Supplement: Supplementary file 1 — Additional file 1: Fig. S1. (A) Fluorescence spectra of UCMPs (a) and UCMPs@MIL-100 (b); (B) fluorescence spectra of UCMPs@MIL-100@NIP (a), UCMPs@MIL-100@MIP before (b) and after (c) extraction; (C) UV–Vis spectra of β-LG (a) and fluorescence spectra of UCMPs@MIL-100@MIP (b); Fig. S2. Fluorescent spectra of UCMPs@MIL-100@MIP without protein in 20 ℃ and 44 ℃; Fig. S3. (A) Optimization of UCMPs@MIL-100 dosage, (B) addition ratio, and (C) the pH of the adsorption system; Fig. S4. Adsorption kinetics of UCMPs@MIL-100@MIP and UCMPs@MIL-100@NIP to β-LG; Fig. S5. Correlation curve of the results between standard HPLC and the prepared fluorescence sensor. [file 12951_2022_1258_MOESM1_ESM.docx]

**Supporting Information**

**A UCMPs@MIL-100 based thermo-sensitive molecularly imprinted fluorescence sensor for effective detection of β-lactoglobulin allergen in milk products**

**Liping Hong, Mingfei Pan^*^, Xiao Yang, Xiaoqian Xie, Kaixin Liu, Jingying Yang, Shan Wang, Shuo Wang^*^**

State Key Laboratory of Food Nutrition and Safety, Tianjin University of Science & Technology, 300457 Tianjin, China; honglpstu@163.com (L. Hong); yangx2021@126.com (Xiao Y.); qianxx8135@163.com (X. Xie.); Liukx2019@163.com (K. Liu.); yangjy0823@126.com (J. Yang); wshan0929niu@163.com (S. Wang)

*Corresponding author: Mingfei Pan and Shuo Wang

Tel: (86 22) 60912493

Fax: (86 22) 60912493

E-mail: pmf2006@sina.com.cn, [s.wang@tust.edu.cn](mailto:s.wang@tust.edu.cn)


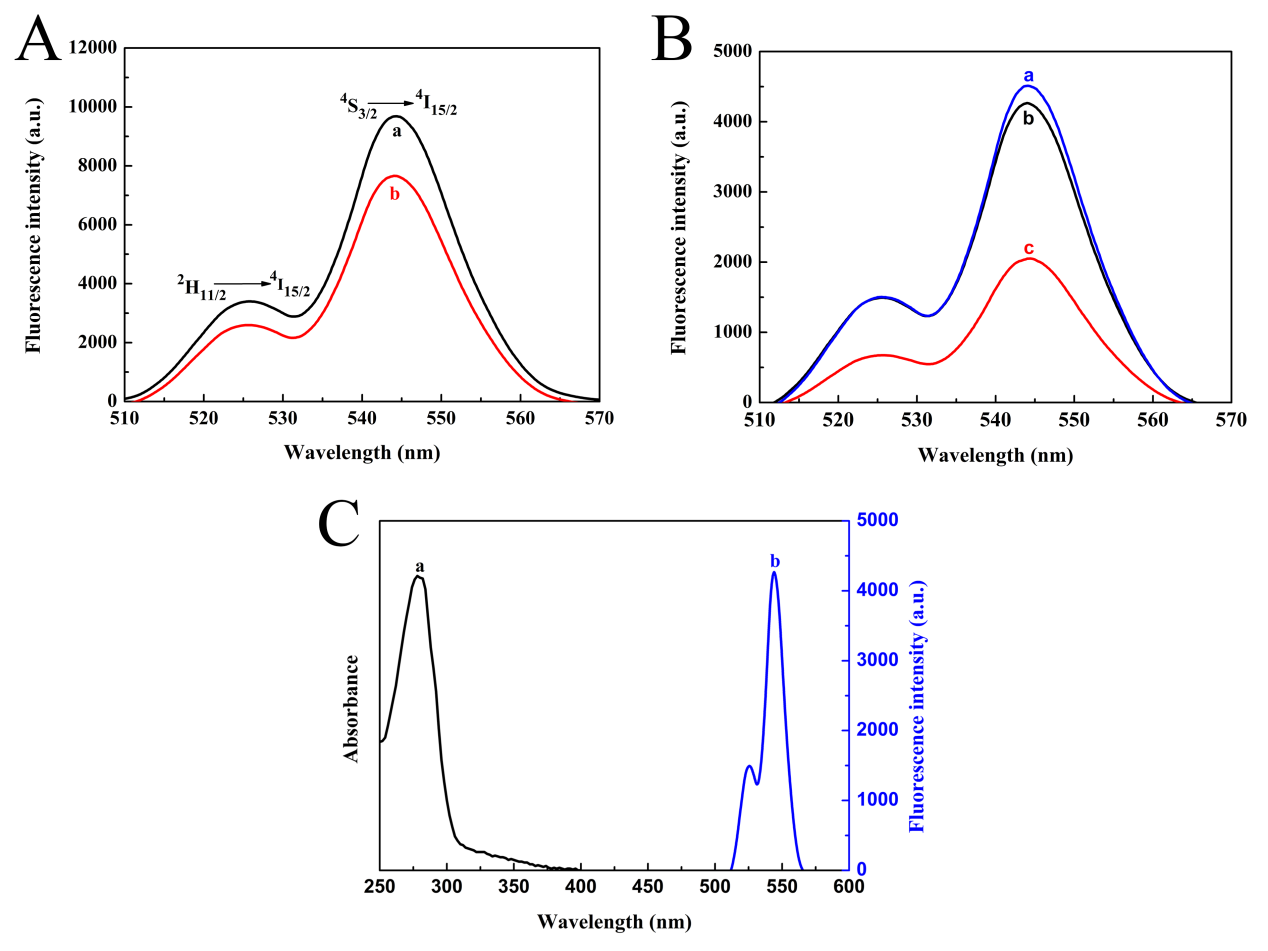


Fig. S1 (A) Fluorescence spectra of UCMPs (a) and UCMPs@MIL-100 (b); (B) fluorescence spectra of UCMPs@MIL-100@NIP (a), UCMPs@MIL-100@MIP before (b) and after (c) extraction; (C) UV-Vis spectra of β-LG (a) and fluorescence spectra of UCMPs@MIL-100@MIP (b).

**
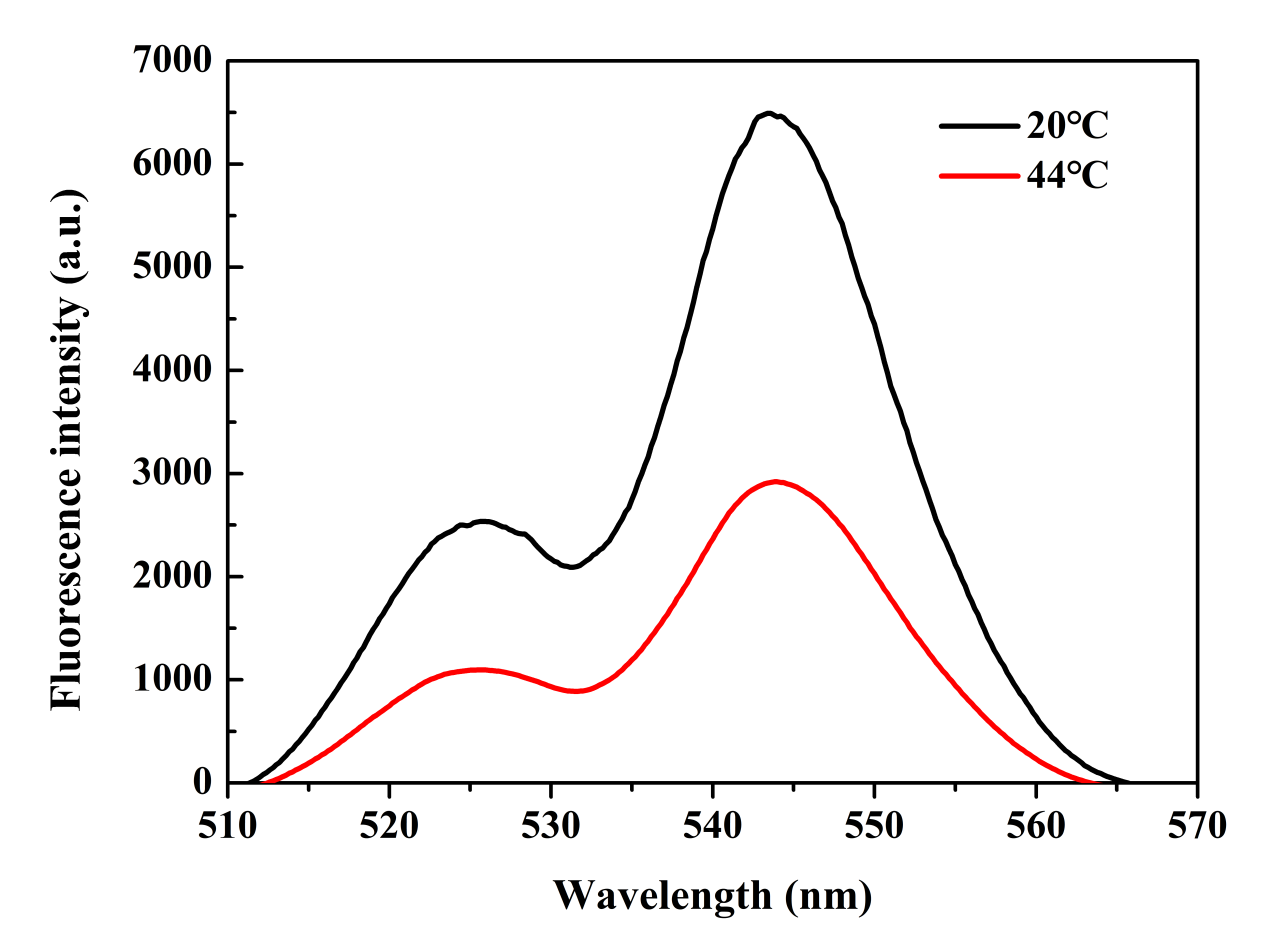
**

Fig. S2 Fluorescent spectra of UCMPs@MIL-100@MIP without protein in 20 ℃ and 44 ℃.


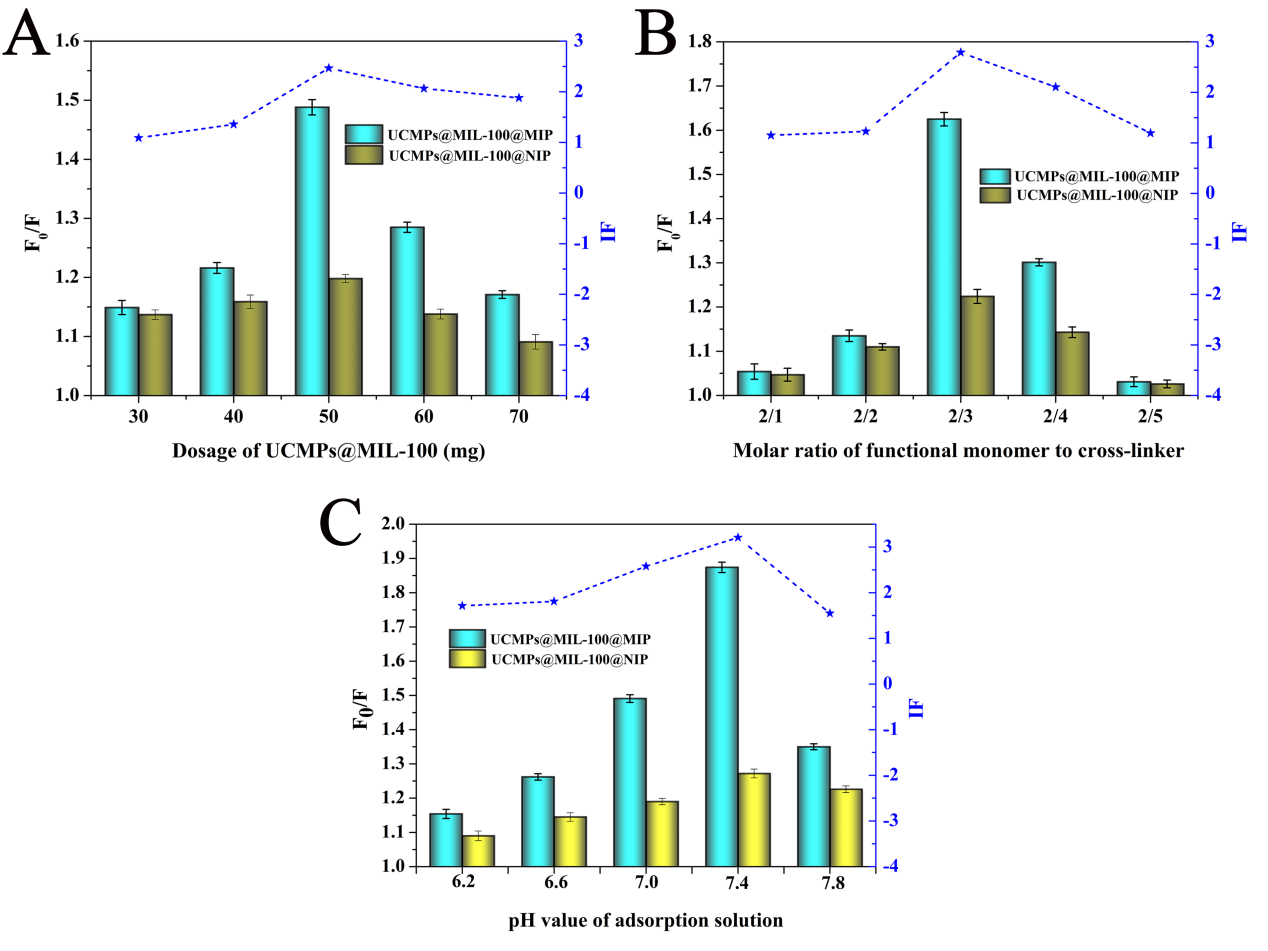


Fig. S3 (A) Optimization of UCMPs@MIL-100 dosage, (B) addition ratio, and (C) the pH of the adsorption system.


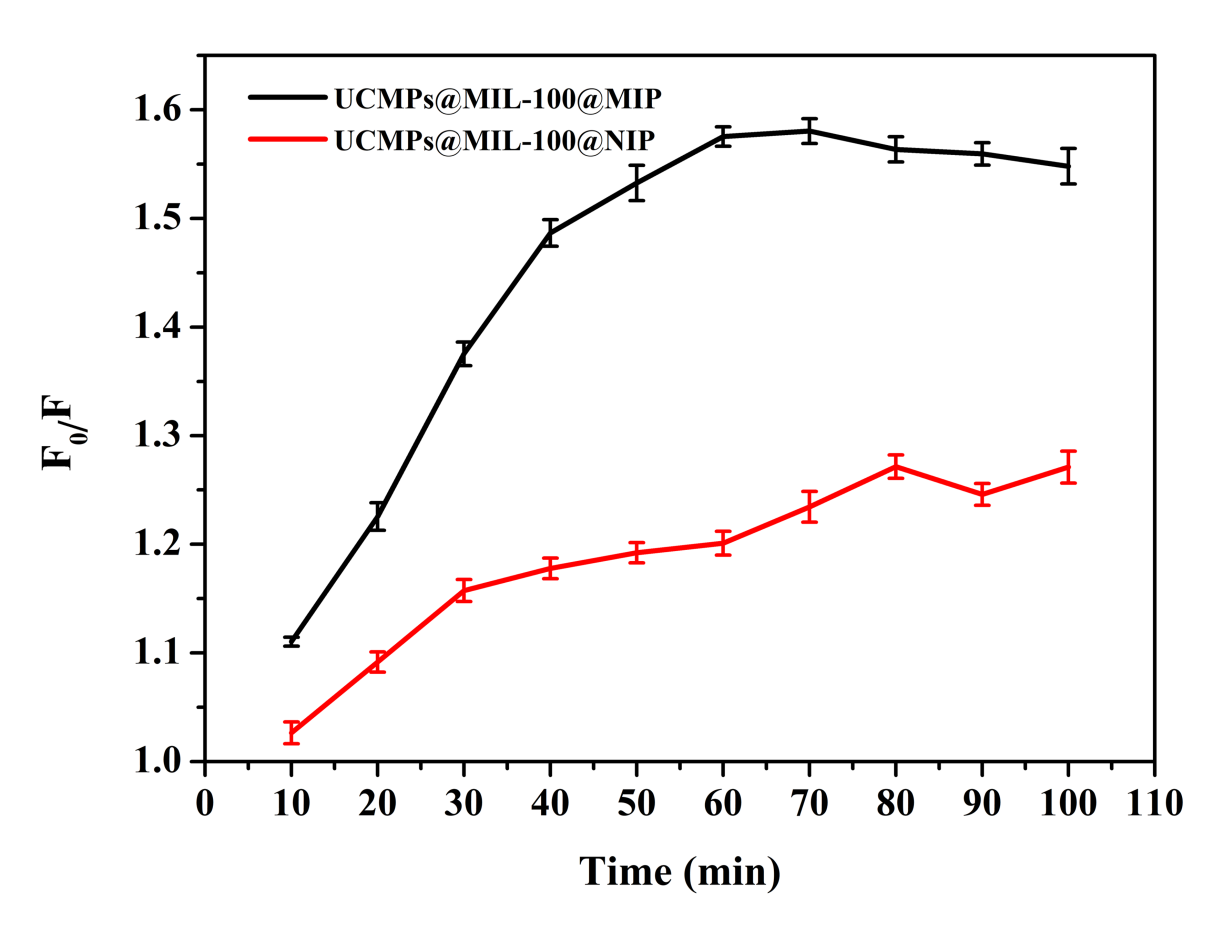


Fig. S4 Adsorption kinetics of UCMPs@MIL-100@MIP and UCMPs@MIL-100@NIP to β-LG


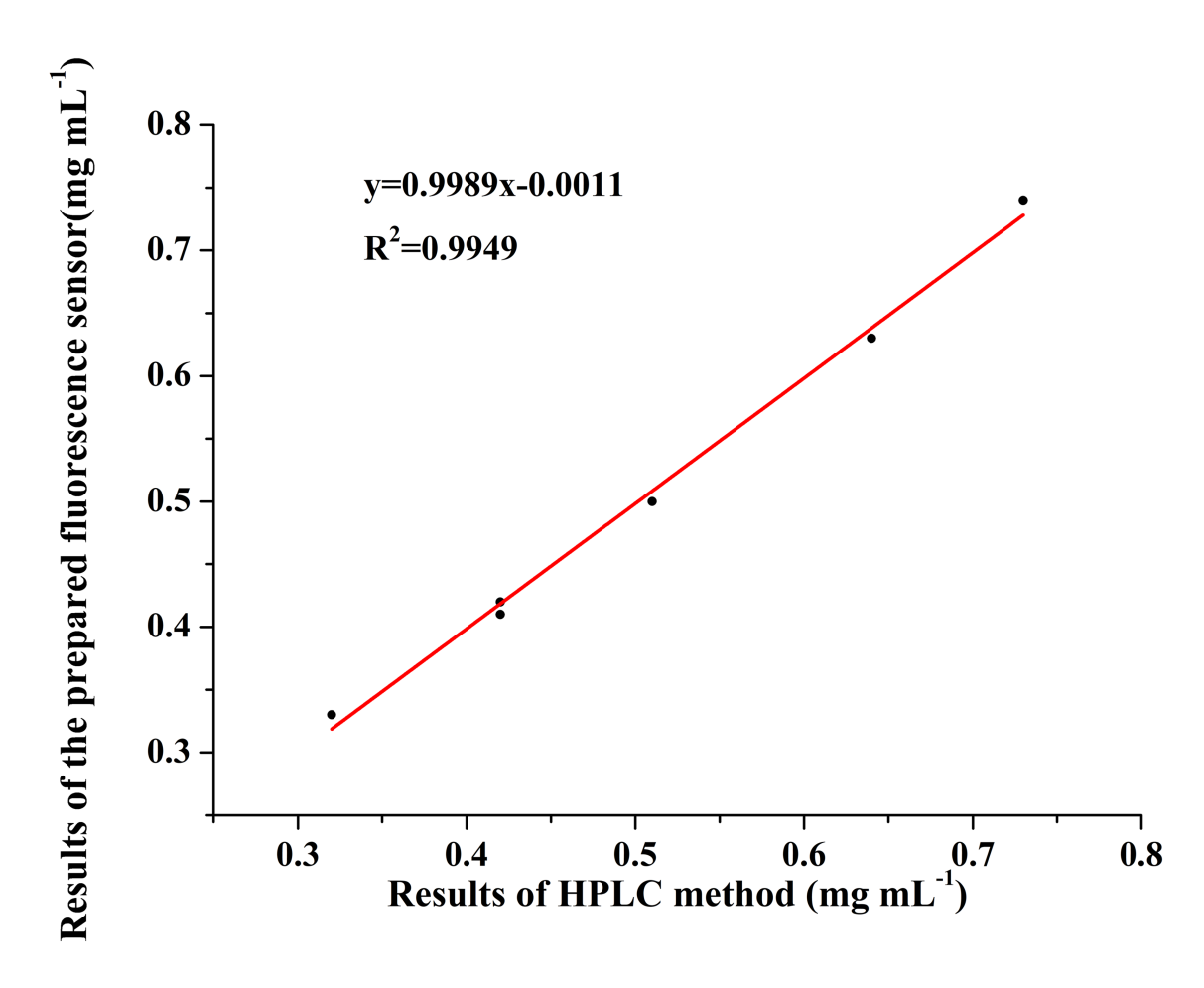


Fig. S5 Correlation curve of the results between standard HPLC and the prepared fluorescence sensor.
